# Effectiveness of IV Cannulation Skills Laboratory Training and Its Transfer into Clinical Practice: A Randomized, Controlled Trial

**DOI:** 10.1371/journal.pone.0032831

**Published:** 2012-03-12

**Authors:** Frederike Lund, Jobst-Hendrik Schultz, Imad Maatouk, Markus Krautter, Andreas Möltner, Anne Werner, Peter Weyrich, Jana Jünger, Christoph Nikendei

**Affiliations:** 1 Department of General Internal Medicine and Psychosomatics, University of Heidelberg Medical Centre, Heidelberg, Germany; 2 Department of Nephrology, University of Heidelberg, Heidelberg, Germany; 3 Department of Psychosomatic Medicine and Psychotherapy, University of Tübingen, Tübingen, Germany; 4 Department of Diabetes, Endocrinology, Angiology, Nephrology and Clinical Chemistry, University of Tübingen, Tübingen, Germany; University of York, United Kingdom

## Abstract

**Background:**

The effectiveness of skills laboratory training is widely recognized. Yet, the transfer of procedural skills acquired in skills laboratories into clinical practice has rarely been investigated. We conducted a prospective, randomised, double-blind, controlled trial to evaluate, if students having trained intravenous (IV) cannulation in a skills laboratory are rated as more professional regarding technical and communication skills compared to students who underwent bedside teaching when assessed objectively by independent video assessors and subjectively by patients.

**Methodology and Principal Findings:**

84 volunteer first-year medical students were randomly assigned to one of two groups. Three drop-outs occurred. The intervention group (IG; *n* = 41) trained IV cannulation in a skills laboratory receiving instruction after Peyton's ‘Four-Step Approach’. The control group (CG; *n* = 40) received a bedside teaching session with volunteer students acting as patients. Afterwards, performance of IV cannulation of both groups in a clinical setting with students acting as patients was video-recorded. Two independent, blinded video assessors scored students' performance using binary checklists (BC) and the Integrated Procedural Protocol Instrument (IPPI). Patients assessed students' performance with the Communication Assessment Tool (CAT) and a modified IPPI. IG required significantly shorter time needed for the performance on a patient (IG: 595.4 SD(188.1)s; CG: 692.7 SD(247.8)s; 95%CI 23.5 s to 45.1 s; p = 0.049) and completed significantly more single steps of the procedure correctly (IG: 64% SD(14) for BC items; CG: 53% SD(18); 95%CI 10.25% to 11.75%; p = 0.004). IG also scored significantly better on IPPI ratings (median: IG: 3.1; CG: 3.6; p = 0.015;). Rated by patients, students' performance and patient-physician communication did not significantly differ between groups.

**Conclusions:**

Transfer of IV cannulation-related skills acquired in a skills laboratory is superior to bedside teaching when rated by independent video raters by means of IPPI and BC. It enables students to perform IV cannulation more professionally on volunteer students acting as patients.

## Introduction

The teaching of scientific knowledge, basic clinical skills and moral values is essential for medical students in order to develop medical professionalism [1]. Traditionally, the triad of knowledge, skills and attitudes [2] required for medical practice has been imparted in the course of bedside teachings in a hospital ward [3]. Providing an opportunity for the demonstration of clinical procedures through teachers as well as their observation and ultimately independent performance by students, ward-based teaching and medical clerking essentially draws upon the instructional principle of “see one, do one” [4]. Despite the fact that bedside teaching has always been deemed an indispensable and valuable method of teaching [5], [6], its role is declining in medical schools [7]. Technological, economic and regulatory changes have led to a considerable reduction in bedside teaching opportunities for medical students and young doctors [8].

Practising on real patients is a problematic ethical issue, particularly where it involves the training of invasive procedures [9]. In addition, the quality of ward-based training is often impaired by lack of supervision through physicians and frequent assignment of students to routine activities of limited educational value [10], [11]. Basic clinical skills acquisition on wards occurs in a rather “haphazard” fashion and frequencies of performance of such skills differ widely among students [10]. Two recent studies [12], [13] showed that up to a quarter of American third-year medical students and up to a fifth of American medical fourth-year students never had performed basic clinical skills such as phlebotomy, arterial blood sampling and peripheral IV catheter insertion. Yet, mastering these procedures is considered to be essential for medical students [14]. In order to bridge the gap between expectations and actual learning experiences on the wards, structured and “proficiency-based” [15] teaching interventions, such as skills training sessions in simulators, have been increasingly integrated into medical curricula over the past decades [8], [16].

Today, skills laboratories have taken on a central role in the training of procedural skills such as IV cannulation, urethral catheter insertion or physical examination. Skills laboratory training allows for sustained deliberate practice [17] in a “mistake forgiving” [18], safe environment. It can be structured and standardized employing different instructional approaches such as Rodney Peyton's “Four-Step Approach” [19], [20] or “Mental Training” [21]. At the end of each session, students are provided with educational feedback, an inherent feature of simulation based medical education (SBME) [8], [22], enabling them to reflect on their performances. In skills laboratories medical students commonly train clinical procedures among each other [23], together with Standardised Patients (SPs) [24] or by using manikins or (part)-task-trainer models [25].

The effectiveness of medical skills laboratories has been demonstrated by several works and is widely recognized. In a systematic review, Lynagh et al. [26] concluded that skills laboratory training enhances procedural skills performance compared to standard or no training when assessed by simulator performance. On undergraduate educational level, skills laboratory training leads to better results in written skills tests [27] and to improved performance of basic clinical skills in OSCEs [28], [29]. This effect can be demonstrated regardless of whether the preceding skills laboratory training is lead by faculty staff or by trained medical students that serve as peer teachers [30], [31]. Yet, the transfer of procedural skills acquired in skills laboratories to actual clinical practice remains the subject of an ongoing discussion. Remmen et al. assumed that skills training provides a better preparation for clinical clerkships [32] and leads to an increase in the number of clinical procedures performed on ward [33]. Neither of the studies, however, assessed the actual quality of skills performed on patients. Frequently, a pressing need for well-designed studies to further investigate this topic of “translational research” [34] has been enunciated [22], [26]. Lynagh et al. reported that only 20 out of 44 studies, that were included in their review, respond to the question, if improvements in performance on medical simulators in fact translate to improved clinical performance. Eight of these 20 studies, however, used animal models to demonstrate the transfer. The remaining twelve studies that assessed the transfer of simulator performance to clinical performance on real patients, focused on postgraduate training of either endoscopic or laparoscopic surgery skills only, with many of them being weakened by methodological flaws such as small sample size or failure to report on the method of randomisation or blinding technique.

Up till today, there is an apparent lack of evidence of the transfer of skills laboratory training of procedural skills on undergraduate medical educational level. We therefore conducted a prospective, randomised, controlled, double-blind trial to answer the question: Are students having received a training of intravenous (IV) cannulation in a skills laboratory setting perceived and rated as more professional regarding technical and communication skills compared to students who underwent bedside teaching when assessed 1) objectively by independent video assessors and 2) subjectively by patients?

## Methods

### Trial design

**Figure 1:**
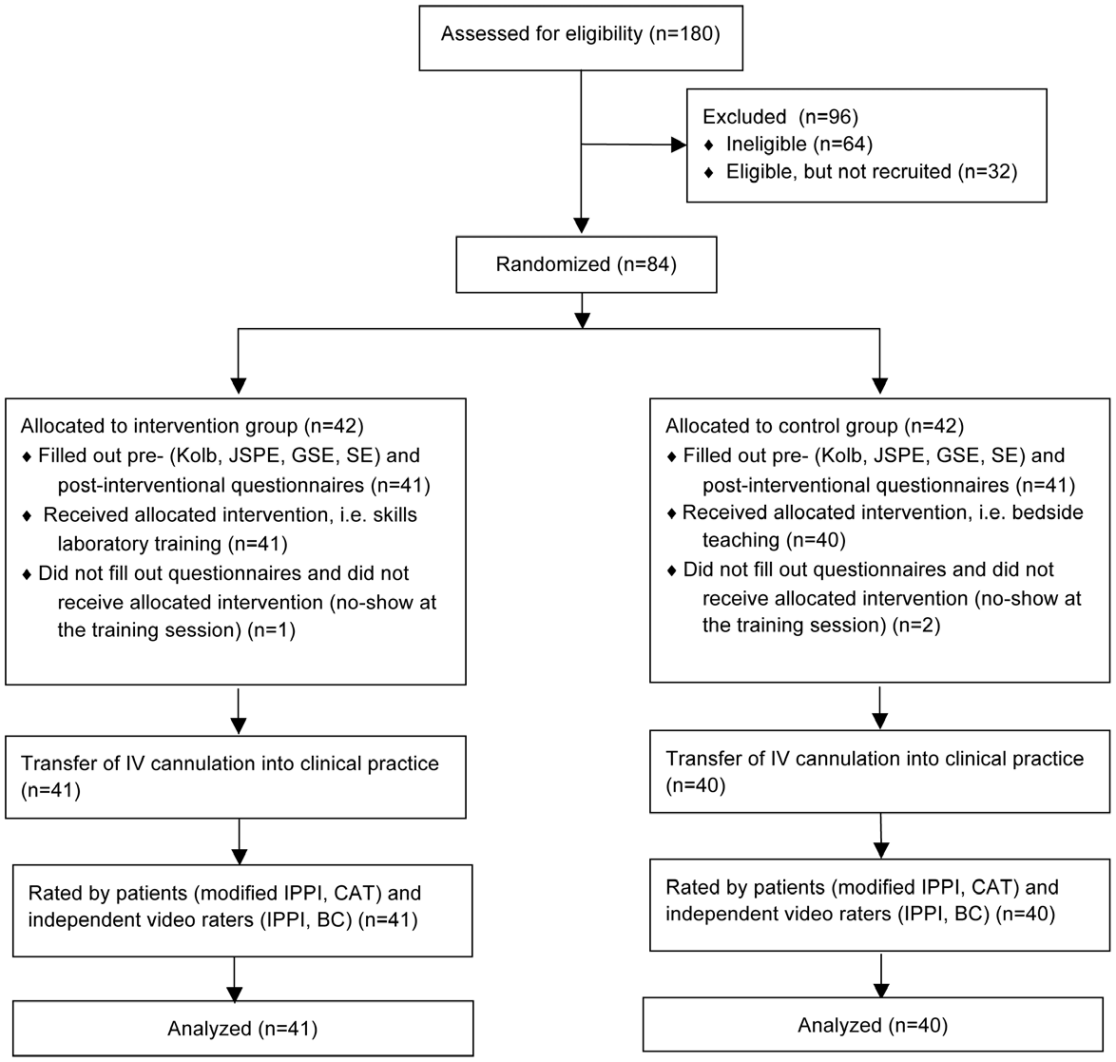
Study design. * Kolb = Kolb Learning Style Inventory; JSPE = Jefferson Scale of Physician Empathy; GSE = General Self-Efficacy; SE = IV cannulation related Self-Efficacy. Post-interventional Evaluation = Evaluation of teaching model acceptance. IPPI = Integrated Procedural Protocol Instrument; CAT = Communication Assessment Tool; BC = Binary Checklist. Figure modified from the CONSORT 2010 flow diagram templates [50], [51].

We conducted a prospective, randomized, controlled, double-blind clinical trial to investigate the transfer of skills laboratory training of procedural skills at undergraduate medical education level (see Checklist S1). For this purpose 84 first-year medical students of the University of Heidelberg, Germany were randomly assigned to one of two groups. The intervention group (IG) trained IV cannulation in a skills laboratory. The control group (CG) took part in a bedside teaching on IV cannulation with volunteer students acting as patients. Following the skills laboratory training or bedside teaching, acceptance of skills laboratory training and bedside teaching was evaluated by students. Subsequently, performance of IV cannulation of both groups in a clinical setting with volunteer students acting as patients was video-recorded. Student performance was assessed by independent video assessors and students who acted as patients (see Figure 1).

### Participants

Student sample. Recruitment of participants took place among first year medical students within their first week of term. All first-year medical students enrolled at Heidelberg University in the winter term of 2009, aged 18 to 30 years, who agreed to participate were included into the study prior to the randomization procedure. Criteria for exclusion from the study were previous training as a paramedic or nurse and previous experience in performing technical procedures such as taking blood samples, IV cannulation or intramuscular injections. Information about these exclusion criteria was obtained by means of a questionnaire, which was handed out to the students within the first week of term.

Additionally, group characteristics (baseline data) such as age (years), sex (female/male), previous education in a healthcare related or medical profession other than paramedic or nurse (i.e. physiotherapy, dental assistant, etc.), completed nursing electives and civil service (i. e. “compulsory paid community service”, which represented an alternative to military service in Germany until suspension of conscription in 2011) were obtained by means of a questionnaire.

#### Patient sample

With regard to the reduced availability of patients on the wards and the fact, that IV cannulation of a patient constitutes an invasive clinical procedure, which always requires an indication, volunteer students acting as patients were recruited for this study. This decision was also made to ensure the practicability of the planned study. Accordingly, 84 volunteer students were recruited among second- and third-year students.

Criteria for exclusion from participation in the study were intake of coagulation-inhibiting medication and/or regular intake of steroids, known existence of a chronic infection such as hepatitis B, C or HIV or state of acute illness. Volunteer students were allowed to refuse disclosure of personal details regarding the above mentioned chronic infections. In this case, however, a participation in the trial was no longer possible. Volunteer students, who were regular smokers, who suffered from coagulopathies and/or diabetes, and/or had received organ transplantation were also excluded from the study. All criteria were applied in order to minimize risks of potential harm for both, students and volunteer students acting as patients. All volunteer students were blinded to the study design and the participants' allocation to the intervention or control group. In addition, 14 fifth-year volunteer students were recruited to act as patients in the bedside teaching sessions of the CG. These 14 fifth-year volunteer students were used for demonstration purposes by the physician only. All 95 volunteer students, who acted as patients in the study received a written role-play instruction as well as detailed written information on the purpose of the study.

### Acquisition of data

The trial was conducted over a three-week period at the beginning of term at the University of Heidelberg, Germany. Data were collected on the premises of the Department of Internal and Psychosomatic Medicine at Heidelberg University Hospital.

### Ethics

Ethics approval was granted by the ethic committee of the University of Heidelberg (Nr. S-211/2009). Written consent was obtained from all participants. Study participation was voluntary and all candidates were assured of anonymity and confidentiality.

### Pre-Interventional Questionnaires for Participating Students

Students were characterized by means of the following standardized questionnaires: The Kolb Learning Style Inventory (LSI) [35], [36], the Jefferson Scale of Physician Empathy (JSPE) [37] and the General Self-Efficacy Scale (GSE) [38]. Additionally, IV catheter insertion-related ratings of self-efficacy were obtained.

The Kolb Learning Style Inventory was used to investigate the different learning styles among students. It is built upon the theory of experiential learning [35] and is designed to define an individual's specific learning preference. Kolb [36] assumed that the process of grasping new information and experience was based on two dialectally related modes: Concrete Experience (CE) - Abstract Conceptualization (AC), and Reflective Observation (RO) - Active Experimentation (AE). The LSI consists of 12 items that ask respondents to rank four sentence endings that refer to the four above mentioned different learning modes. [36] Ranking is done on a four-point Likert scale (4 = “most like me” to 1 = “least like me”).

The Jefferson Scale of Empathy [37] assesses empathy among physicians, medical students and health professionals as a multidimensional concept. Since the assessment of students' IV cannulation skills after the intervention included ratings of their empathy towards the patient during the procedure, the Jefferson Scale was used to assess these empathy skills pre-interventionally within the student sample. The scale consists of 20 items that are answered on a seven-point Likert scale. Ten of these items with positive factor structure coefficients are directly scored on a scale ranging from 1 = “strongly disagree” to 7 = “strongly agree”. An example of a directly scored item is: “A physician who is able to view things from another person's perspective can render better care.” The other ten items have large negative factor structure coefficients and are reverse scored on a scale ranging from 7 = “strongly disagree” to 1 = “strongly agree”. An example of a reverse-scored item is ‘Emotion has no place in the treatment of medical illness.’ A higher score on the scale indicates greater empathy. [37]

The General Self-Efficacy Scale was designed to assess perceived self-efficacy in the event of adversity and stressful life events [38]. It consists of ten items, that are each rated on a four-point Likert scale (1 = “I agree” to 4 = “I disagree”). The GSE was used to investigate the students' self-efficacy and motivation prior to the intervention.

IV catheter insertion-related ratings of self-efficacy were obtained from all students before the teaching sessions by means of a questionnaire that contained five statements on the topic of IV catheter insertion, e.g. “I am able to insert an IV catheter on a patient”. These statements were rated on a six-point Likert scale (1 = I completely agree to 6 = I completely disagree).

### Interventions

#### Skills laboratory training

The intervention group (*n* = 41) trained IV cannulation in a skills laboratory receiving instruction according to Rodney Peyton's ‘Four-Step Approach’ [19], [20]. The fourth step, i. e. the first self-dependent performance on the manikin, was only performed once by each student of the IG in this study. Lessons were held for groups of three students with a teacher ∶ student ratio of 1 ∶ 3. Emphasis was placed on the self-contained practical exercise of peripheral IV catheter insertion on a part-task-trainer model in the shape of a human arm (serial number: AN 1121; name: “Training Arm Adult Venipuncture and Injection- White”, purchased via Erler & Zimmer from Nasco – Modesto, CA, USA). The part-task-trainer model allows for the puncture of multiple veins, e.g. the cephalic vein, the basilic vein and the median cubital vein. The exercise was carried out as a role-play to create a more realistic training situation, to enhance the students' involvement and to support the acquisition of patient-physician communication [23], [39]. For this purpose, students obtained detailed role-play instructions from the teacher. Teachers as well as students were dressed in white coats during the teaching session. Following the practical exercise, all students received feedback through the teacher. Time of instruction was recorded.

#### Bedside teaching

The control group (*n* = 40) received bedside teaching on IV cannulation based on the instructional principle of “see one, do one”. This principle represents a traditional teaching approach in which clinical skills are first demonstrated and explained by the trainer and then performed independently on a patient by the trainee himself. [4], [20] Other authors speak of “the traditional experience-based model” [15] or “the apprenticeship model” [3] when referring to a concept in medical education where “students practice new skills on patients”. [15] In the control group, no specific training other than watching the physician and listening to his explanations was provided prior to the students' first independent performance of IV cannulation on a volunteer student acting as a patient. Lessons were held for groups of three students with a physician ∶ student ratio of 1 ∶ 3. The experienced ward physicians demonstrated insertion of a peripheral IV line on students, who volunteered as patients. The physicians were supposed to act as role models for the correct technical execution of the procedure, for the patient-physician communication and for the adequate care of the patient's needs. Students were asked to watch the demonstration attentively. Ward physicians as well as students were dressed in white coats during the teaching session. No flip-charts, black boards or computers were used to visualise the contents of teaching. Time of instruction was recorded.

#### Skills laboratory teachers and ward physicians

Teachers consisted of experienced skills laboratory teachers for IG and experienced ward physicians for CG. Teachers of the intervention group participated in a pre-interventional briefing on skills laboratory training according to Peyton's “Four-Step Approach” [19], [20]. Skills laboratory teachers and ward physicians received a detailed manual in order to prepare themselves. This manual included defined learning goals, a comprehensive teaching agenda and detailed information about the time available for each section of the teaching session. All skills laboratory teachers and ward physicians were blinded to the study design and were only involved in either teaching IG or CG.

### Post-Interventional questionnaires for participating students

Post-intervention, students were asked about their valuation of the teaching session. This was done to assess the students' acceptance of the different teaching models and to investigate the students' perception of their teachers' and physicians' motivation and didactic competency. Five statements about the teaching modalities, the motivation of the teachers and the feedback, that was given, were rated on a six-point Likert scale (1 = “I completely agree” to 6 = “I completely disagree”).

### Outcomes

#### Transfer of learning outcomes

Following the intervention, performance of IV cannulation of each student of both groups was video-recorded in a clinical setting with volunteer students acting as patients. IV cannulation took place in the Department of Internal and Psychosomatic Medicine of Heidelberg University Hospital. During cannulation, the students acting as volunteer patients were seated in a room which included a bed, chair and table and was designed after the ward rooms of Heidelberg University Hospital in order to simulate a clinical environment. All participants had a maximum of three attempts of IV cannulation. The total amount of time needed for a finally successful cannulation was recorded. After two unsuccessful attempts with the volunteer students acting as patients, students had to perform the third attempt on a part-task-trainer model due to ethical aspects.

### Assessment of trained skills

#### Video rating

Performance of participants was video-taped in both IG- and CG-groups by means of high-resolution cameras with optical zoom to capture all details necessary for an exact evaluation. Videos were digitally processed and randomized in order to render any potential inference on students' group identity impossible. In the following, two blinded video assessors evaluated the performance of the students independently using a binary checklist [40] and the global rating form of the Integrated Procedural Protocol Instrument (IPPI), proposed by Kneebone et al. [41] for the assessment of procedural skills in a clinical context. The video assessors were experienced internal medicine consultants with wide experience of teaching and assessing clinical skills and special training in assessing students by means of checklists. The binary checklist [40] consisted of 25 items that listed the number of procedural steps needed to insert a peripheral IV catheter. Items on the list were ticked as soon as they were demonstrated correctly by the student during at least one attempt. A six-point Likert scale (1 = “very good” to 6 = “unsatisfactory”) was used for global IPPI ratings [41], that consisted of 11 items. IPPI ratings [41] were done in terms of an “overall impression” after each student had finished their total amount of attempts needed for IV cannulation. The IPPI [41] item “Overall ability to perform the procedure [including technical and professional skills]” was used for clinical validation of the students' performance. For this purpose, the absolute number of “competent students” (students, who received ‘1’ and ‘2’ ratings), “borderline students” (‘3’ and ‘4’ ratings) and “incompetent students” (‘5’ and ‘6’ ratings) with regard to their technical competence and professionalism while performing the task was calculated.

#### Time and number of attempts needed for successful IV cannulation

The time needed for successful IV cannulation was measured in seconds, using the processed video material. The number of attempts needed for successful cannulation was counted. An attempt of cannulation was defined as successful once the catheter was inserted and placed correctly in the vein and was properly supported with a madrain and adhesive dressing.

#### Patient rating

Students' performance was assessed by patients by means of a modified Communication Assessment Tool (CAT) [42] and a modified global rating form on the basis of the IPPI [41]. For study purpose, twelve of the 15 items of the CAT were considered (1–2, 4–10, 12–14). The CAT is a reliable and valid instrument that can be used by patients to assess interpersonal and communication skills of physicians [42]. The eleven items of the IPPI [41] were modified to first person statements, e.g. “Assessment of patient's needs before procedure.” was changed into “The doctor assessed my needs before he started with the procedure.” A six-point Likert scale (1 = “very good” to 6 = “unsatisfactory”) was used for both, CAT and IPPI [41] ratings.

### Sample size

A power analysis revealed that *n* = 42 students were needed for each study group in order to detect an expected effect size of 0.6 SD for the rating form of the Integrated Procedural Protocol Instrument (IPPI) [41] (*α* = 0.05; power 0.8). Our calculations were based on the work of Weyrich et al. [30] on peer-assisted learning in skills laboratory training. Weyrich et al. also assessed IV cannulation skills of medical students. Consequently, 84 volunteer first-year medical students were randomly assigned to one of two groups. Due to the absence of three students at the training session, a total of 81 students ultimately participated in the study (IG: n = 41; CG: n = 40). Consequently, three volunteer students, who were supposed to act as patients for these three first-students, had to be excluded from participation.

### Randomization procedure

Students were allocated according to a 1∶1 ratio to either skills laboratory training or bedside teaching (IG: *n* = 42; CG: *n* = 42) by means of blocked randomization using a fixed block size of six. The randomization sequence was created by a person with no clinical involvement in the trial using computerized random numbers. Stratification was used to balance subjects with regard to the factor “gender” (female, male). Strict confidentiality regarding the block size was maintained. The allocation sequence was concealed from the students and video raters, who were responsible for assessing the participants, as well as from the researchers, who were involved in the statistical testing of the data (CN, AM).

### Blinding

All participants, including students and volunteer students acting as patients, all teachers of the skills laboratory sessions, all ward physicians and the two independent video raters were blinded to the study design and the students' allocation to either intervention or control group. Participants were only informed about that the purpose of the study was to investigate two different teaching models. No details about the different teaching models were provided.

### Statistical analysis

Data are presented as means ± standard deviation (SD) or medians. Normally distributed nominal data such as age, years of medical studies, time and attempts needed for IV cannulation, length of the teaching session and binary checklist results were compared using a Student's t-test after being tested for equal variances (non-significant Levene's test). A Mann-Whitney U-Test (MWU) was used for ordinal data such as the pre- and post-interventional questionnaires for participating students, IPPI [41] and CAT ratings [40]. Distribution of group characteristics (baseline data) referring to sex, preceeding education in a healthcare related or medical profession other than paramedic or nurse (i.e. physiotherapy, dental assistant, etc.), completed nursing electives and civil service were compared by chi-square-tests. Interval level data was tested for normal distribution and skewness. All data are presented as absolute numbers and percentages or mean and standard deviation. For the readers' convenience, results of the MWU tests are displayed as medians and not as sum of ranks. A *p*-value<0.05 was considered to be statistically significant. Effect sizes were calculated using Cohen's d. Standardized inter-rater reliability for the two video assessors was calculated based on residual maximum likelihood (REML) estimates of variance components. Raw data was processed using Microsoft EXCEL. The software package STATISTICA (Statsoft, Inc, Tulsa, OK, USA) was used for statistical analysis.

## Results

### Participant flow

See Figure 1 for a detailed diagram. Due to the absence of three students at the training sessions, a total of 81 students (IG: *n* = 41; CG: *n* = 40) eventually participated in the study and were analysed.

### Baseline data

#### Student sample

All 84 students who were recruited for the study were first-year medical students. The mean age was 19.9 SD(1.8) years (IG) and 20.4 SD(2.5) years (CG; p = 0.277), respectively. Both study groups consisted of 16 male and 26 female participants. There were no significant differences detected in any of the group characteristics listed in Table 1. Additionally, no significant differences were shown for the results of the standardized questionnaires LSI [35], [36], JSPE [37], GSE [38] and the results of the IV catheter insertion-related self assessment expectations that were used to characterize the participants pre-interventionally (see Table 1).

**Table 1:** Baseline data.

	IG (n = 42)	CG (n = 42)	p
**Age (years)**	19.86 (1.80)	20.38 (2.53)	0.277
**Gender (female)**	26 (61.9%)	26 (61.9%)	1.000
**Gender (male)**	16 (38.1%)	16 (38.1%)	1.000
**Preceeding eduaction in a health care related or medical profession**	2 (2.4%)	1 (1.2%)	0.557
**Civil service**	10 (23.8%)	8 (19.0%)	0.595
**Nursing elective**	28 (66.7%)	30 (71.4%)	0.637

	IG (n = 41)	CG (n = 40)	p
	Median	Median	
**Kolb LSI (10–40)**			
__Abstract Conceptualization	35	36	0.967
__Concrete Experience	23	22	0.951
__Active Experimentation	32	34	0.836
__Reflective Observation	29	30	0.402
**JSPE (20–140)**	115	114.5	0.936
**GSE (40–10)**	21	21.5	0.336
**Pre-Interventional IV cannulation-related Self-Efficacy (1–6)**	5.4	5.4	0.354

LSI = Kolb Learning Style Inventory; JSPE = Jefferson Scale of Physician Empathy; GSE = General Self-Efficacy. Data are means (SD) or numbers (%) or medians. P-values were calculated using students t-test for age, chi-quadrat test for gender, preceeding health care related or medical profession other than paramedic or nurse (i.e. physiotherapy, dental assistant, etc.), civil service and nursing electives. Mann-Whitney U-Tests for LSI, JSPE, GSE and Pre-Interventional IV cannulation-related Self-Efficacy.

#### Patient sample

The mean age of volunteer students acting as patients was 23.1 SD(2.8) (patients of IG) and 22.5 SD(2.1) (patients of CG); p = 0.277. On average, patients were at the beginning of their third year of studies. About one half of the volunteer students acting as patients had already performed phlebotomy (patients of IG: 22.62%; patients of CG: 17.86%; p = 0.374) and/or IV catheter insertion (patients of IG: 28.57%; patients of CG: 22.62%; p = 0.275) themselves during a clinical elective or in the course of professional training as a paramedic or nurse prior to studying medicine.

#### Skills Laboratory Teachers and Ward Physicians

Skills laboratory teachers and ward physicians were comparable in age and years of work experience with an overall average of 33.8 SD(6.2) years of age (IG: 34.2 SD(8.4); CG: 33.4 SD(3.9); p = 0.852) and 5.3 SD(5.1) years of work experience as medical doctors (IG: 6.3 SD(6.4); CG: 4.3 SD(3.8); p = 0.556). Both IG and CG had two female and three male skills laboratory teachers or ward physicians, respectively.

#### Length and acceptance of teaching sessions

Length of teaching sessions did not significantly differ between groups (IG: 74.3 SD(11.7) min; CG: 65.9 SD(11.3) min; p = 0.065), yet there was a difference of 8.4 minutes of teaching time on average between the two groups. Acceptance of teaching sessions varied among students. Participants of the CG showed a tendency to find their bedside teaching sessions more realistic (IG: 2.29 SD(1.12), CG: 2.15 SD(1.45) p = 0.088). After the session, however, students of the IG felt significantly more confident of being able to memorize the learning contents in particular (IG: 1.50 SD(0.68); CG: 2.00 SD(0.99), p = 0.032) as well as significantly more motivated regarding the insertion of an IV catheter on their own (IG: 1.49 SD(0.64); CG: 2.13 SD(1.20), p = *0.019*). Additionally, IG stated that they benefited significantly more from the feedback given by the skills laboratory teachers compared to the CG (IG: 1.39 SD(0.54); CG: 2.40 SD(1.75), p = *0,016*).

### Video rating

#### Number of attempts and total amount of time needed

Both study groups needed 2.4 SD(0.8) attempts on average for the insertion of an IV catheter (IG 2.39 SD(0.81); CG: 2.43 SD(0.84); 95% CI 0.0 to 0.1; p = 0.850). Students of the IG, however, made significantly more successful attempts than students of the CG (IG: 36; CG: 26; p = *0.015*). Additionally, IG needed a significantly shorter time for the performance on a patient (IG: 595.4 SD(188.1)s; CG: 692.7 SD(247.8)s; 95% CI 23.5 s to 45.1 s; p = *0.049*).

#### IPPI ratings

**Figure 2:**
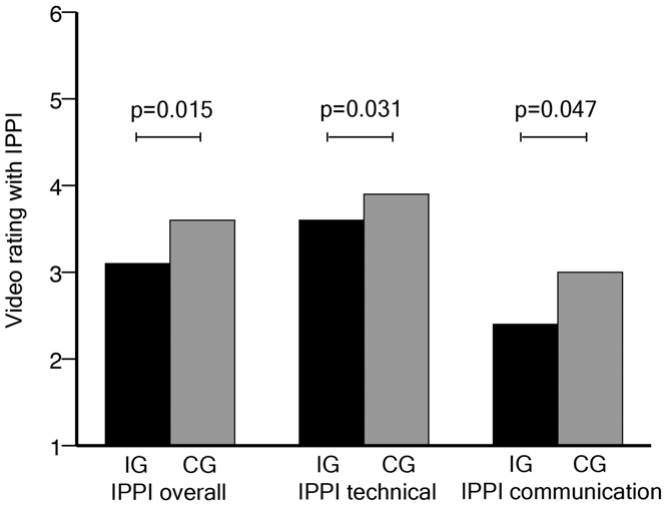
IPPI ratings presented according to the teaching model employed. *Columns represent median scores. Over-all IPPI ratings (items 1–11). Technical skills ratings (items 4–6; 9–11). Communication skills ratings (items 1–3; 7; 8). P-values calculated based on group-comparisons (Mann-Whitney U-Test).

IG scored significantly better on IPPI [41] ratings (median scores: IG: 3.1; CG: 3.6; p = *0.015*) as shown in Table 2. After dividing the IPPI's eleven items [41] into items which describe “technical skills used during the procedure” (4–6; 9–11) as well as items which mainly characterize “communication skills used during the procedure” (1–3; 7; 8), a sub analysis of these two categories was carried out. Students of the IG also received significantly better ratings in these sub-categories (median scores: IG: 3.6; CG: 3.9; p = *0.031* for technical skills; IG: 2.4; CG: 3.0; p = *0.047* for communication skills) as shown in Table 2 and Figure 2.

**Table 2:** Results of the video ratings of students' IV cannulation skills.

	IG (n = 41)	CG (n = 40)		
	Mean (SD)	Mean (SD)	SEM_D_ (95% CI)	p
**Binary Checklist (%)**	64 (14)	53 (18)	11 (10.25 to 11.75)	*0.004*
**Time needed for IV cannulation (sec)**	595.4 (188.1)	629.7 (247.8)	34.3 (23.5 to 45.1)	*0.049*
**Attempts needed for IV cannulation (1–3)**	2.39 (0.81)	2.43 (0.84)	0.04 (0.0 to 0.1)	0.850

	Median	Median	p
**IPPI: Overall (1–6)**	3.1	3.6	*0.015*
**IPPI: Technical (1–6)**	3.6	3.9	*0.031*
**IPPI: Communication (1–6)**	2.4	3.0	*0.047*

BC = binary checklist (percent of correctly demonstrated procedural single steps); IPPI = Integrated Procedural Performance Instrument (11 items, Likert-scale rating ranging from 1 = very good to 6 = unsatisfactory); SD = standard deviation; SEM_D_ = standard error of the mean difference; P-values are calculated with students T-test results for BC and Mann-Whitney U-Test results for IPPI.

In view of the underlying “nested design” of the actual study (three students per teaching session, IG = 14 groups; CG = 14 groups), statistical analysis showed that the groups of the IG still received significantly better IPPI ratings [41] compared to the CG when comparing means of groups (median scores: IG: 3.15; CG: 3.47; p = 0.016).

Although the expected effect size of 0.6 SD as mentioned above in the sample size calculation could not be detected for IPPI ratings [41], the detected effect size of 0.3 SD is still considered to be small to medium.

Using the IPPI item “Overall ability to perform the procedure [including technical and professional skills]” [41] as a way of clinical validation, nine IG students were rated as “competent”, 24 as “borderline”, and eight as “incompetent”, while in the CG seven students were regarded to be “competent”, 14 to be “borderline”, and 19 to be “incompetent”.

#### Binary checklist

The number of accurately performed steps by a student was calculated as a percentage of the total amount of steps identifiable on the video tape. Due to camera angles and limited abilities to zoom, some items listed on the 25-item binary checklist could not be identified in the video recordings of a minority of students' performances (items identifiable of 25 items in total: Rater 1: IG: 24.78 SD(0.42); CG: 24.33 SD(1.37); Rater 2: IG: 24.39 SD(0.74); CG: 24.05 SD(1.68)). No significant difference was detected as to the number of identifiable items between the two different study groups.

Students of the IG completed significantly more single steps of the procedure correctly (IG: 64% SD(14) for binary checklist items; CG: 53% SD(18); 95% CI 10.25% to 11.75%; p = *0.004*), as listed in Table 2. Statistical analysis in consideration of a “nested design” showed, too, that groups of the IG completed significantly more single-steps correctly (IG: 64 SD(11) percent of binary checklist items; CG: 54 SD(14), p = *0.047*).

### Patient-rating

Rated by volunteer students acting as patients, students' procedural performance and patient-physician communication did not differ significantly between groups (p = 0.544 for CAT [42]; p = 0.683 for modified IPPI ratings [41]) as shown in Table 3.

**Table 3:** Results of the patient-ratings of students' IV cannulation skills.

	IG (n = 41)	CG (n = 40)	
	Median	Median	p
**CAT (1–6)**	1.9	2.0	0.544
**IPPI: Overall (1–6)**	2.6	2.5	0.683
**IPPI: Technical (1–6)**	2.5	2.8	0.205
**IPPI: Communication (1–6)**	2.6	2.2	0.576

CAT = Communication Assessment Tool (12 items, Likert-scale rating ranging from 1 = “very good” to 6 = “unsatisfactory”); IPPI = Integrated Procedural Performance Instrument (11 items, Likert-scale rating ranging from 1 = “very good” to 6 = “unsatisfactory”); median; P-values were calculated using Mann-Whitney U-Test results.

### Inter-rater reliability

Standardised inter-rater reliability was 0.910 (p = 0.001) for binary checklists [40] and 0.734 (p = 0.001) for the global rating form of the IPPI [41]. Regarding a sub analysis of the items of the IPPI [41], inter-rater reliability was 0.717 (p = 0.001) for “technical skills used during the procedure” and 0.697 (p = 0.001) for “communication skills used during the procedure”.

## Discussion

This study prospectively investigated the effectiveness and transfer of IV cannulation skills acquired by undergraduate medical students in the course of a skills laboratory training session compared to medical students who underwent bedside teaching with volunteer students acting as patients. For this purpose students' technical and communication skills were assessed objectively by independent video assessors and subjectively by patients by means of binary checklists and global rating forms. Prior to the intervention, students were carefully selected according to predefined criteria of in- and exclusion. No significant differences in relevant socio-demographic variables were detected between the two study groups. Students who had received training of IV cannulation in a skills laboratory setting, scored significantly better in binary checklist ratings and on global IPPI ratings [41]. Practising IV cannulation in a skills laboratory setting also resulted in a significantly shorter time needed for the performance on a patient and students of the intervention group demonstrated significantly more successful attempts of IV catheter insertion. Rated by patients, students' procedural performance and patient-physician communication did not significantly differ between the two study groups.

The superior results of binary checklist ratings [40] and IPPI global rating forms [41] indicate, that students, who had trained IV cannulation in a skills laboratory not only performed significantly more single-steps of the procedure correctly, but also were perceived as more professional in terms of technical and communication skills compared to students of the control group. The detected effect size for IPPI ratings [41] was small to medium.

The differences in checklists and global rating forms between the intervention group and the control group seem to indicate a directly observable difference in the students' clinical performance particularly when regarding the results of the clinical overall rating, which are reflected in the last item of the IPPI [41] “Overall ability to perform the procedure (including technical and professional skills)”: Decisively more students, who were trained in a skills laboratory setting, were rated as being clinically “borderline” compared to the bedside teaching group, whereas “incompetent” students were by far less frequent. These clinical ratings validate the observed effects in this study.

Up till today, only few studies have dealt with the transfer of procedural skills acquired in medical skills laboratories. These studies primarily refer to the performance of endoscopic or laparoscopic surgery skills compared to standard or no training. [26] In a comparative meta-analysis McGaghie et al. [43] showed only recently that SBME with “deliberate practice” [44] is superior to traditional clinical medical education in the acquisition of laparoscopic surgery skills, advanced cardiac life support, cardiac auscultation skills, hemodialysis catheter and central venous catheter insertion skills and thoracentesis skills. In our study, we demonstrated that even basic procedural skills such as IV cannulation can be successfully transferred from a simulated setting into clinical practice. Referring to this, our results show that training IV cannulation in a skills laboratory enhances the quality of the procedure performed on a patient and simultaneously leads to a decrease in the amount of time needed for the procedure in comparison to bedside teaching.

In a BEME systematic review, Issenberg et al. [8] outlined simulator validity and feedback as key features of medical simulations, “that lead to most effective learning”. These features may also have contributed to the observed results in our study. In this study, validity of the skills laboratory training was improved by means of role-play. Role-playing enhances the realism of technical skills training and leads to better patient-physician communication during the sessions as demonstrated by Nikendei et al. [39] for doppler-sonography and gastric tube insertions training sessions. Regarding feedback, it is little surprising that the students, who had trained IV cannulation in the skills laboratory, stated that they benefited significantly more from the feedback given by the skills laboratory teachers compared to the control group. Since the students of the control group did not perform IV cannulation on their own before their first attempt on a patient, they did not receive specific feedback about their own performance. The importance of feedback for effective learning was demonstrated by Ericsson et al. [45] and emphasized by Issenberg et al. [8] and McGaghie et al. [22] for the concept of SBME.

Furthermore, the method of instruction seems to play an important role in the course of effective learning [20]. The fact that the intervention group required significantly shorter time for a superior performance of IV cannulation on a patient, implicates, that the intervention group was able to recapitulate the procedure's single steps faster and more accurately. This corresponds with the students' statement after the skills laboratory teaching sessions, who said that they felt significantly more capable to memorize the learning contents in particular compared to the control group. This effect might be explained by the known advantage of Peyton's “Four-Step Approach” [19] compared to a standard instruction, which has only recently been demonstrated by Krautter et al. [20]. In this study, students who had undergone skills laboratory training of gastric tube insertion according to the “Four-Step Approach” [19], scored significantly better on IPPI ratings [41] and global communication rating scales than students who had received skills laboratory training with a standard instruction method. When asked to perform gastric tube insertion self-dependently on a manikin, the intervention group needed, comparable to our study, significantly less time for the performance. The authors attributed this effect in particular to the third step of Peyton's “Four-Step Approach”, which is thought to facilitate memory consolidation through the concept of “motor imagery” [46], [47]. Contrary to our study, however, the transfer of gastric tube insertion into clinical practice was not investigated by Krautter et al. [20].

Our results are validated by reliability measures of binary checklist ratings and global rating forms obtained in the objective video rating. Both instruments showed a high to very high inter-rater-reliability. In line with the literature, inter-rater reliability was higher for binary checklist ratings than for global IPPI ratings [41]. This confirms that checklists allow for a more standardized and more reliable evaluation of the technical performance of a student [48], whereas global ratings constitute a more summative measure [40]. Global rating scales are superior in measuring higher levels of clinical competence, expertise and professionalism [40], [49].

Interestingly, students' procedural performance and patient-physician communication did not significantly differ between the two study groups when rated by students acting as patients. This effect might be explained by the fact that – contrary to the video raters – our “patients” were neither specifically trained, nor were they experienced raters. More importantly though, since each student who acted as a patient encountered only one student during the study, the “patients” were unable to compare their personal experience of having an IV cannula inserted into their veins with the performance of other students. Additionally, based on their personal experience with IV cannulation as medical students, the students acting as patients may have rated the students' performance more benevolently than real patients: It might have reminded them of their own struggles and fears when they had to insert an IV line into a patient's arm for the first time.

### Limitations

Several limitations of this study should be mentioned. This study assessed the effectiveness of IV cannulation skills training and its transfer into clinical practice only. We assume that our results can be applied to technically related techniques which require venipuncture, such as taking blood samples or blood cultures. The generalisability of the results with regard to less related techniques should be evaluated by future studies.

All 84 students, who took part in the study, were volunteers. As such they might have had a different disposition, for example in terms of motivation or enthusiasm towards certain training models than compared to the population at large.

Since the control group was not allowed to practise IV cannulation prior to the first self-dependent procedure on a patient, the superior results of the intervention group seem unsurprising. This methodological approach, however, is consistent with the clinical reality of “see one, do one”. In order to ensure that every student received the same amount of attention from the teacher, we arranged for the duration of the teaching session and the teacher : student ratio to be the same in both study groups.

Due to the post-test design of this study, no conclusion can be made as to the participants' performance of IV cannulation before the intervention. In light of the study's exclusion criteria, the participants' minimal medical knowledge and low pre-interventional self-efficacy, it can be assumed, that the students' abilities to perform IV cannulation were equally limited. A pre-post-test design would not have been suitable due to ethical reasons with uninstructed students performing IV cannulation on patients. In addition, a pre-interventional capture of the students' IV cannulation performance would already have been a training of this skill.

After two unsuccessful attempts, students had to perform the third try on a part-task-trainer model due to ethical aspects. Our video raters, however, assessed students' performance globally as an overall rating of all attempts needed.

In this study, third-year medical students acted as patients, who, based on their own experience with IV cannulation, may have rated students' performance differently compared to real patients.

Students, as well as volunteer students acting as patients were aware of being videotaped, which might have influenced their behaviour to some degree. Due to limited camera angles and low definition, raters encountered difficulties in identifying some of the details of the 25 items listed on the binary checklist. However, we consider this problem to be negligible, since the amount of identifiable items was equally high for both raters.

Due to the lack of validated German versions of the IPPI [41] and the CAT [42], we used self-translated versions of the instruments. German translations of the IPPI [41], however, have been used in several internationally published studies [20], [30] on the acquisition of clinical skills, and interrater-reliability was high for both instruments in our study.

Finally, although the group sample size of *n* = 42 was sufficient to measure the effect of skills laboratory training compared to bedside teaching when rated by independent video raters, it may possibly have failed to detect more marginal differences.

### Conclusions

In summary, the results of our study showed that training of IV cannulation skills acquired in a skills laboratory is superior to bedside teaching when rated by independent video raters by means of IPPI scales and binary checklists. Skills laboratory training enables students to perform IV cannulation faster, more accurately and more professionally on students acting as patients in terms of technical and communicational aspects than bedside teaching. These results can be attributed to the didactic approach of the skills laboratory training session in combination with the students' possibility of practising IV cannulation independently in a safe environment before the first actual performance on a patient. Training IV cannulation in a skills laboratory thus leads to an improvement in patient safety and better medical care. Our results underline the importance of an implementation of structured and proficiency-based teaching interventions for the training of invasive procedures on all levels of difficulty, not only in residency programmes, but also in undergraduate medical education programmes. Future studies should address the durability of skills acquired through simulation based training with long-term follow-ups of participants. Furthermore, researchers should investigate how concomitant clinical supervision affects the transfer of procedural skills from a simulated setting into clinical practice.

## Supporting Information

Checklist S1 CONSORT 2010 checklist.
(PDF)
